# First known COVID-19 case and contact tracing efforts in İstanbul, Turkey

**DOI:** 10.3906/sag-2103-30

**Published:** 2021-08-30

**Authors:** Abdullah Emre GÜNER, Aral SÜRMELİ, Kemal KURAL, Esra ŞAHİN, Perihan ALKAN, Erdoğan KOCAYİĞİT, Mustafa HATİPOĞLU, Şuayip BİRİNCİ, Kemal MEMİŞOĞLU, Işıl MARAL

**Affiliations:** 1 Department of Public Health Services, İstanbul Health Directorate, İstanbul Turkey; 2 Medical Rescue Association of Turkey, İstanbul Turkey; 3 Department of Infectious Diseases, Istanbul Health Directorate, İstanbul Turkey; 4 Ministry of Health, Government of Turkey, Ankara Turkey; 5 Istanbul Health Directorate, İstanbul Turkey; 6 Department of Public Health, Medeniyet University School of Medicine, İstanbul Turkey

**Keywords:** Infections, contact tracing, public health

## Abstract

**Background/aim:**

COVID-19 has now become a global pandemic. Understanding the routes of transmission is vital in the mitigation and suppression of the disease. İstanbul has become one of the disease’s epicenters. This study aims to describe the first COVID-19 case and contact tracing efforts around it in İstanbul.

**Materials and methods:**

The descriptive study was conducted in İstanbul, Turkey. The first COVID-19 cases and those associated with them were investigated with contact tracing, and primary and secondary cases were described.

**Results:**

The source case was an individual who returned to Turkey from international travel at the beginning of March and tested PCR (–). The index case is the brother of the source case and is considered the first PCR (+) case diagnosed in İstanbul. Contact tracing revealed 23 PCR (+) cases, 14 of which resulted in hospitalization and three deaths.

**Conclusions:**

This study described cases of the first COVID-19 cluster in İstanbul. Moreover, contact tracing was used in this first cluster. This contributed to contact tracing algorithms in Turkey.

## 1. Introduction 

COVID-19 is a disease caused by a virus from the family Coronoviridae of viruses first described in the Wuhan region of Hubei State in China in December 2019^1^World Health Organization (2020). Modes of transmission of virus causing COVID-19: implications for IPC precaution recommendations [online]. Website: https://www.who.int/news-room/commentaries/detail/modes-of-transmission-of-virus-causing-covid-19-implications-for-ipc-precaution-recommendations [accessed 15 aug 2020]. The disease is characterized by acute respiratory symptoms, similar to those seen in the MERS and SARS outbreaks, and features human-to-human transmission. Although initially thought to be a zoonotic disease, in which the SARS-Cov-2 virus was transmitted from bats to humans, human-to-human transmission is currently the primary way the disease is transmitted [1].

As of February 15, 2021, the number of COVID-19 cases around the World had exceeded 171 million cases, with more than 3.5 million casualties^2^Wordometer (2021). Coronavirus Live Update [online]. Website: https://www.worldometers.info/coronavirus/ [accessed 31 May 2021]. Although some countries have seemingly begun to recover from the peak incidence, a second wave linked to the loosening of public health regulations remains a significant risk [2]. The first case of COVID-19 in Turkey was identified on March 11, 2020, and the first death due to the disease occurred on March 16^3^Ministry of Health (2020). COVID-19 Bilgilendirme Platformu (in Turkish) [online]. Website: https://covid19.saglik.gov.tr/TR-68443/covid-19-durum-raporu.html [accessed 23 October 2020].

The Turkish Ministry of Health commenced preparations for dealing with an epidemic in January 2020, immediately following reports of an emerging epidemic in Wuhan. Simultaneously, the case definitions for probable and confirmed COVID-19 were set down, and testing and treatment algorithms were developed^4^Ministry of Health (2020). Algoritmalar (in Turkish) [online]. Website: https://covid19bilgi.saglik.gov.tr/tr/algoritmalar [accessed 18 September 2020].


Contact tracing has proven essential in mitigating and suppressing the epidemic. Contact tracing allows an index case to be identified and permits the discovery of those at risk of infection so that they may be isolated. It is one of the cornerstones of mitigation and suppression efforts in public health responses to an epidemic. Previous studies concerned with the first cases in particular cities and the associated contact tracing efforts have revealed that person-to-person transmissibility is high for COVID-19, but further research into understanding the attack rates of local outbreaks is called for [3]. 

With a registered population of 15,462,452, İstanbul is the most populous city in Turkey, accounting for almost 19% of the total population^5^Turkish Statistical Institute (2020). Adrese Dayali Nufus Kayit Sistemi Sonuclari (in Turkish) [online]. Website: https://data.tuik.gov.tr/Bulten/Index?p=Adrese-Dayali-Nufus-Kayit-Sistemi-Sonuclari-2020-37210 [ accessed 15 May 2021] . In addition to its central role in the commercial and economic life of the country, İstanbul is home to one of the busiest airports in the World, accommodating 200,000 passengers daily^6^İstanbul Airport (2020). Facts and Figures [online]. Website: https://www.istairport.com/en [accessed 18 August 2020]. İstanbul has become evolved into a hotspot of the pandemic.

**Figure 1:**
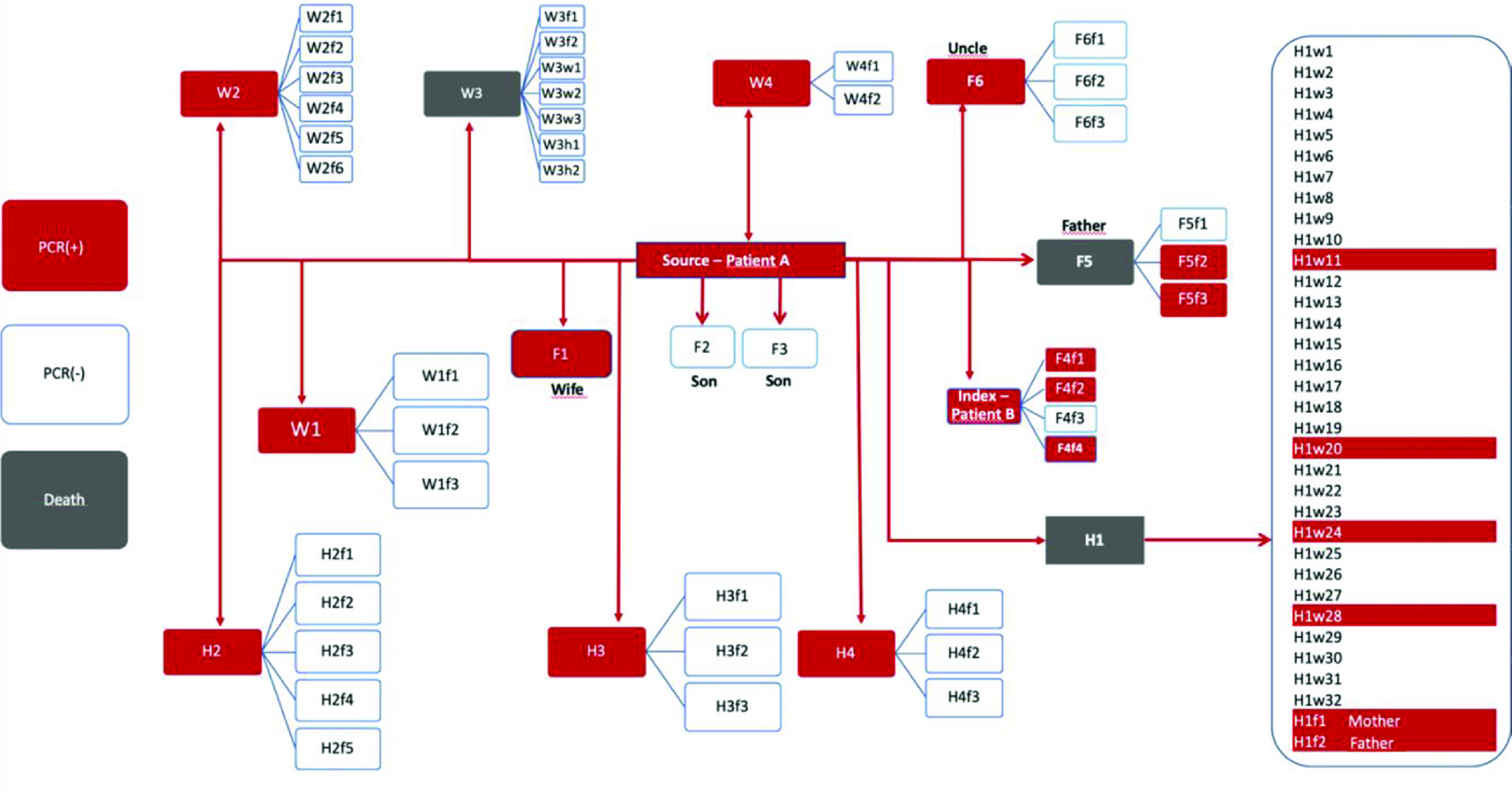
Contact tracing map of index and source cases.

This study describes the first COVID-19 case and contact tracing around the index case in İstanbul, Turkey.

## 2. Materials and methods

In this article, we describe the first polymerase chain reaction positive (PCR (+)) COVID-19 case (index case), together with both suspected and confirmed cases discovered by tracing their contacts. We outline the clinical characteristics of the index case and examine disease progression in the suspected or confirmed cases of COVID-19 amongst the contacts of the index and source case. 

The relevant laboratory test results, symptoms, follow-up, and treatment protocols were obtained from the Ministry of Health’s national COVID-19 Surveillance system database. At the same time, contact tracing reports were acquired from the İstanbul Public Health Directorate. Tracing reports were reevaluated by a public health official, and a field contact tracer was allocated to the specific case. No additional data collection was undertaken for the purposes of this study. The İstanbul Medeniyet University Göztepe Research and Training Hospital Internal Review Board granted ethical approval via decree number: 2020/0208 for the study.

### 2.1. Diagnosis and treatment of COVID-19 cases in Turkey

Turkey employs the WHO’s case definition, which is regularly updated. Any person presenting with symptoms that may suggest a COVID-19 infection (cough, fever, shortness of breath) is advised to apply to a hospital for COVID-19 testing. A PCR test is used to confirm cases of COVID-19. If COVID-19 is confirmed, the patient is admitted to the hospital or recommended to self-isolate at home. In the latter situation, the patient is followed up by daily phone calls. 

All PCR (+) cases, plus those diagnosed clinically but without a PCR (+) result, start treatment according to the most recent version of the treatment protocol designed by the Turkish Ministry of Health’s COVID-19 Scientific Committee, a committee of clinicians and public health officials appointed to advise on and prepare the strategies for diagnosis and treatment of COVID-19 plus any public health interventions required. All diagnoses and treatments related to COVID-19 are provided free of charge to all citizens, even in private hospitals. 

### 2.2. Contact tracing

When a PCR (+) case is identified, notification is sent to the local Public Health Directorate and the patient’s registered general practitioner to facilitate contact tracing. Field teams call and visit all possible contacts for symptom surveillance and PCR test sampling. Patients are followed up via phone every day until the point where symptoms have not been present for 14 days, and are advised to self-isolate during this period. In the contacts of a patient who develops symptoms, the usual COVID-19 case diagnosis and treatment protocols are used. 

### 2.3. Index case

An index case is defined as the first patient identified with PCR-confirmed COVID-19. The source case is then the individual who brought and transmitted the disease from an international source. Primary cases are described as confirmed cases of COVID-19 who are in close contact with the source or index case, and for whom no other confirmed or suspected case of COVID-19 is possible as a source of the illness [4].Secondary cases are confirmed cases of COVID-19 patients who are believed to have been infected by the primary cases identified by the contact tracing investigation [4]. 

In this study, the index case is coded as Patient B, and the source case as Patient A. Contact tracing for both cases was undertaken simultaneously since after the international trip of Patient A, they spent time together and had contact with the same people. Contacts of the index case or source (primary contacts) and their contacts (secondary contacts) are categorized into ‘family’, ‘work place’, and ‘hospital’ with ‘F’, ‘W’, and ‘H’ as the respective designations. The contacts of the index and source cases are coded with uppercase letters, while the secondary contacts are coded with lowercase. The codes are also numbered in the order of being traced. Contact mapping is presented in a separate figure (Figure 1).

The age, comorbidity, duration of symptoms, and hospital stay are indicated for all hospitalized patients and all patients with a PCR (+) test. 

## 3. Results

Eight days postreturn from overseas travel, Patient A presented in hospital with fatigue, fever, and cough. A PCR test was performed, which was evaluated as a negative result. Patient B developed symptoms 1 day after Patient A. Patient B is the sibling of Patient A. They had been traveling together for business. Patient B, however, was found to be PCR (+) for COVID-19. Thus, Patient B is the index case in İstanbul. Contact tracing was performed simultaneously for both cases, as the contacts for both individuals were the same after Patient A returned from overseas. 

**Figure 2:**
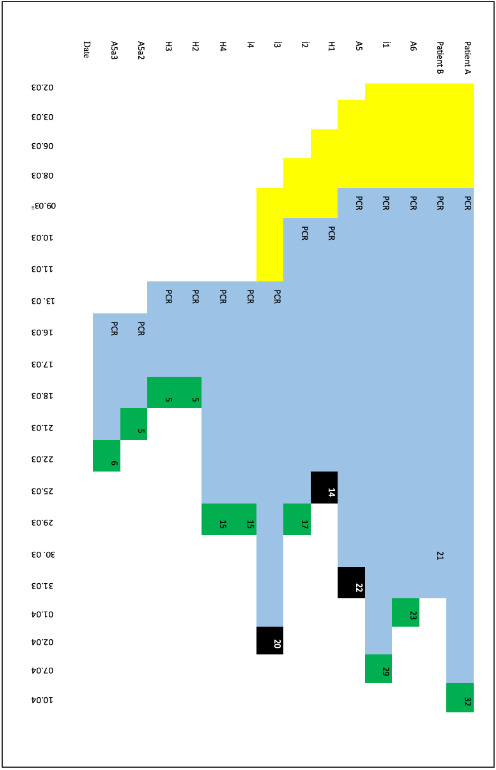
Length of symptoms and hospital admission of hospitalized patients.

The index case was admitted to the hospital, where he remained for 21 days in total, 16 of which were in the ICU (intensive care unit); he was intubated for 10 days (Figure 2). He was admitted on the third day of symptoms, i.e. on day 12. 

Patient A was retested 15 days after his initial (negative) PCR test. This time, the test was positive. Despite COVID-19 being diagnosed with PCR earlier in Patient B than in patient A, Patient A is considered the source case since he had a history of overseas travel compatible with becoming infected, unlike Patient B. Patient A remained in the hospital for 32 days but was neither admitted to ICU nor required intubation. The ages and comorbidities of all contact-traced persons are presented in Table 1. Neither Patient A nor Patient B had any comorbidities or other known risk factors, such as older age. 

**Table 1:** Prognosis of COVID-19 PCR-positive cases.

Cases	Age	Comorbidity	Hospitalization duration	ICU duration	Intubation duration	Prognosis
Patient A (source)	39	-	32	None	None	Recovered
Primary cases		-				
F1	30	-	None	None	None	Asymptomatic
Patient B (index)	34	-	21	16	10	Recovered
F5	66	HTN, DM	22	21	21	Deceased
F6	60	-	23	20	2	Recovered
W1	43	-	29	5	1	Recovered
W2	46	-	17	None	None	Recovered
W3	76	-	20	19	19	Deceased
W4	38	-	15	9	None	Recovered
H1	32	Immunsup.	14	14	3	Deceased
H2	36	-	5	None	None	Recovered
H3	39	-	5	None	None	Recovered
H4	29	-	15	None	None	Recovered
Secondary cases						
F4f1	33	-	None	None	None	Recovered
F4f2	2	-	None	None	None	Recovered
F5f1 Although F5f1 has never turned PCR (+), the patient showed similar symptoms to COVID-19 and was hospitalized.	60	HTN	9	None	None	Recovered
F5f2	81	HTN	5	None	None	Recovered
F5f3	76	-	6	None	None	Recovered
H1f1	55	-	None	None	None	Asymptomatic
H1f2	55	-	None	None	None	Asymptomatic
H1w11	29	-	None	None	None	Asymptomatic
H1w20	50	-	None	None	None	Asymptomatic
H1w24	32	-	None	None	None	Recovered
H1w28	36	-	None	None	None	Asymptomatic
F4f4	29	-	None	None	None	Asymptomatic

There were 12 PCR (+) cases amongst the contacts of the source case. These are the primary cases. Four primary cases were family contacts (F), 4 were workplace contacts (W), and 4 were hospital contacts (H) (Table 2). Three family contacts were admitted to ICU and intubated. Three workplace contacts were also admitted to ICU and intubated. Amongst the H contacts, only one individual was admitted to ICU and intubated (Tables 1 and 2).

**Table 2:** Clinical presentation and course of hospitalization of index case.

Day	1	2	3	4	5	6	7	8	9	10	11	12	…	Day 21
Outpatient				Inpatient					ICU & intubation				Between days 13 and 21, remission in all symptoms and laboratory tests was observed.	Discharge
Fever	+	+	+	+	+	+	+							
Malaise	+		+	+										
Cough			+	+										
Chest Radiography							++	++	+++	++	+	+		Normal
Lab results indicative ofCOVID-19 Laboratory results, excluding CRP levels, were evaluated as a whole. Results outside the normal range of laboratory values are presented in the table.					Leukopenia									
CRP CRP level over 3 mg/dL is marked as ↑, CRP level over 10 mg/dL is marked as ↑↑, any meaningful decrease is marked as ↓.	↑	↑	↑	↑	↑↑	↑	↑	↑	↑	↑	↓	↓		Normal

Three patients, one from each category of contact, suffered a fatal outcome. The hospital contact who died was a 32-year-old woman with immunosuppression. The F contact who died was the 66-year-old father of the source case, while the W contact was 76 years old. All these individuals were admitted to ICU and intubated after being admitted to the hospital. Contact tracing identified 73 contacts of the primary cases (i.e. secondary cases) who were then tested for COVID-19 by PCR, with positive results in 18% of these (n = 13). The mortality among secondary cases was 0% (Figure 1). One of the secondary cases was an F contact. This individual is the mother of the index case (F5f1). However, despite a 9-day admission to the hospital, repeated PCR tests were negative.

While the spouse of the source case tested PCR (+), their children, aged 6 and 8, were negative on PCR testing (Figure 1). 

One patient (H3) tested PRC (+), (–), (+), (–), and (–) consecutively . The positive result on the third test was deemed a false-positive (Figure 2).

The contact tracing investigation of the index case in İstanbul uncovered 12 primary cases confirmed by PCR plus an additional 11 secondary cases confirmed by PCR (n = 23). One further case amongst the secondary contacts was diagnosed with clinical signs without being PCR (+). Three fatalities were observed amongst the primary cases, while there were none in the secondary cases (Figure 2).

Only 14 out of 73 individuals traced from both index and source cases had symptoms on or before the day of investigation. The most common symptom was fever, followed by cough and malaise. Seven out of 23 people were asymptomatic (Figure 1).

The median length of hospital stay was 15 days (IQR: 6–21 days) (Figure 2). For primary contacts of the index or source case, the median length was 17, and for secondary contacts, it was 6 days. The median age of all PCR (+) patients was 39 (IQR: 32–58 and mean 44). The median age of hospitalized patients was 43 years (IQR: 36–66 and mean 50). The deceased patients (n: 3) were aged 32, 66, and 76 years. Four patients admitted had some form of comorbidity. Three were hypertensive, with one also declaring diabetes mellitus. Another individual was suffering from an immunosuppressive disorder. Two of the deceased patients had comorbidities (Table 1).

## 4. Discussion

### 4.1. Main findings of the study

This case report describes the first known cases of COVID-19 in İstanbul, the clinical course of the disease, and the primary and secondary cases arising among their contacts. With a history of international travel, the source case is assumed to have brought COVID-19 into this group, even though he had serial PCR (–) results but turned PCR (+) later and a relatively mild clinical course, without the need for ICU admission. His brother, who had an earlier clinical course and a PCR (+) result in his first test, eventually leading to ICU admission, is designated as the index case of the country.

The distribution of PCR (+) cases seems to be between family, work, and hospital contacts. Deaths also show a similar distribution. Two of the patients who died had some form of comorbidity. While the risk of transmission is believed to be higher with closer and extended contact^7^World Health Organization (2020). Novel Coronavirus – China [online]. Website: https://www.who.int/csr/don/12-january-2020-novel-coronavirus-china/en/ [accessed 18 October 2020], in this cluster, mortality and PCR positivity were similar in all groups (work, family, and hospital), meaning that shorter and less distant contact could also lead to a similar risk of transmission. 

This case study describes importing the first COVID-19 case and cluster into İstanbul and Turkey and transmission from the source and index case. While the start of symptoms to hospitalization for both cases was almost a week, the string of transmission led to 23 confirmed cases in this time frame, underlining the high infectivity of the virus and the need for rapid and comprehensive contact tracing when a COVID-19 case is confirmed. 

There are several limitations to this study. Contact tracing around this case is thought to be near-complete, with all contacts identified. Still, self-declaration by the patients may have led to some of the cases being missed due to fears of stigmatization or marginalization. Besides, since this was a rapidly evolving pandemic, the treatment algorithm in use was being revised almost every week, leading to difficulties in comparing prognoses between cases. 

Contact tracing and the closure of borders happened relatively early in Turkey. Closures started with countries with high traffic and many cases, leading to rapid identification and mitigation of local outbreaks. In addition, contact tracing has been one of the cornerstones of the COVID-19 response in Turkey. In this first cluster, tracers were able to test all the contacts of both index and source cases in a relatively short time to identify the last case of the cluster in a week of investigation. This study described cases of the first COVID-19 cluster in İstanbul with contact tracing. 

## Disclaimers

The opinions expressed by the authors contributing to this article do not necessarily reflect the opinions of the institutions the authors are affiliated with.
